# The socioeconomic distribution of life expectancy and healthy life expectancy in Chile

**DOI:** 10.1186/s12939-023-01972-w

**Published:** 2023-08-22

**Authors:** Manuel Antonio Espinoza, Rodrigo Severino, Carlos Balmaceda, Tomas Abbott, Baltica Cabieses

**Affiliations:** 1 Departamento de Salud Pública, Pontificia Universidad Catolica de Chile, Diagonal Paraguay 362, Piso 2, Santiago, Chile; 2 Unit of Health Technology Assessment, Pontificia Universidad Catolica de Chile, Santiago, Chile; 3 Centro para la Prevención y Control del Cancer, Santiago, Chile; 4 Centro de Salud Global Intercultural, Instituto de Ciencias e Innovación en Medicina, Universidad del Desarrollo, Santiago, Chile; 5 Center for Health Economics, University of York, York, UK

**Keywords:** Health equity, Healthy life expectancy, Socioeconomic disparities in health, Gender equity, Chile

## Abstract

**Background:**

Life expectancy (LE) has usually been used as a metric to monitor population health. In the last few years, metrics such as Quality-Adjusted-Life-Expectancy (QALE) and Health-Adjusted-Life- Expectancy (HALE) have gained popularity in health research, given their capacity to capture health related quality of life, providing a more comprehensive approach to the health concept. We aimed to estimate the distribution of the LE, QALEs and HALEs across Socioeconomic Status in the Chilean population.

**Methods:**

Based on life tables constructed using Chiang II´s method, we estimated the LE of the population in Chile by age strata. Probabilities of dying were estimated from mortality data obtained from national registries. Then, life tables were stratified into five socioeconomic quintiles, based on age-adjusted years of education (pre-school, early years to year 1, primary level, secondary level, technical or university). Quality weights (utilities) were estimated for age strata and SES, using the National Health Survey (ENS 2017). Utilities were calculated using the EQ-5D data of the ENS 2017 and the validated value set for Chile. We applied Sullivan´s method to adjust years lived and convert them into QALEs and HALEs.

**Results:**

LE at birth for Chile was estimated in 80.4 years, which is consistent with demographic national data. QALE and HALE at birth were 69.8 and 62.4 respectively. Men are expected to live 6.1% less than women. However, this trend is reversed when looking at QALEs and HALEs, indicating the concentration of higher morbidity in women compared to men. The distribution of all these metrics across SES showed a clear gradient in favour of a better-off population-based on education quintiles. The absolute and relative gaps between the lowest and highest quintile were 15.24 years and 1.21 for LE; 18.57 HALYs and 1.38 for HALEs; and 21.92 QALYs and 1.41 for QALEs. More pronounced gradients and higher gaps were observed at younger age intervals.

**Conclusion:**

The distribution of LE, QALE and HALEs in Chile shows a clear gradient favouring better-off populations that decreases over people´s lives. Differences in LE favouring women contrast with differences in HALEs and QALEs which favour men, suggesting the need of implementing gender-focused policies to address the case-mix complexity. The magnitude of inequalities is greater than in other high-income countries and can be explained by structural social inequalities and inequalities in access to healthcare.

## Background

The progress in social protection and healthcare observed in the last century has determined a significant improvement in health outcomes. This is often expressed as a remarkable increase in life expectancy (LE) and a reduction of mortality rates of diseases resulting in a higher proportion of people reaching old age [1–3]. However, healthcare interventions such as technological innovations have not only produced increase in the quantity of life but has also impacted on health-related quality of life (HRQoL). Moreover, in some specific cases, the main impact of innovation is upon HRQoL with no major gains in quantity of life [4]. Hence, the idea of examining health impact on these two dimensions is becoming standard in disciplines such as health economics, health services research and public health [5–7].

While LE and HRQoL can be shown separately to monitor population health, it is highly desirable to have one metric capable of integrating both dimensions. The main argument is that people might be willing to trade between these dimensions, for example, they may prefer to enhance their quality of life rather than gaining additional life years. Therefore, the extent to which health is improved depends partially on this trade-off between quantity and quality.

These metrics are generally denominated summary measure of population health (SMPH) [8–10]. The literature provides several SMPHs, which have been developed over the years, each with methodological advantages and disadvantages. In general terms, we can divide them into life years indices and life expectancy indices [9]. Among the former, the most common are disability-adjusted life years (DALYs) and quality-adjusted life years (QALYs). The latter, also called healthy life expectancy (HLE), include disability-free life expectancy (DFLE), disease-free life expectancy, disability-adjusted life expectancy (DALE), health-adjusted life expectancy (HALE), and quality-adjusted life expectancy (QALE) [9]. Among them, QALE and HALE have gained popularity in health research, because their simplicity for interpretation, and their capacity to incorporate quality adjustments using local values - when they have been locally produced and validated. While QALEs are the life years expected at a particular age (e.g., at birth) adjusted by the corresponding quality weight estimated for that age, HALEs expresses the quality adjusted life expectancy, but assuming a progressive decline in HRQoL over time [11].

Another important element related to the measurement of health outcomes is whether to estimate the overall population estimates or their distribution across the population. According to the World Health Organization one of the main objectives of health systems is to achieve a fair health outcome distribution [12]. Notwithstanding, even beyond healthcare, there is strong evidence relating social inequalities and worse population welfare [13–15]. Thus, from a social determinants of health perspective, monitoring the distribution of health is relevant not only to evaluate health system performance, but also the social impact of public policies in general.

Chile is a high-income South American country with a mixed (public and private) healthcare system [16, 17] and pervasive socioeconomic and healthcare inequalities [16–20]. This situation led to include health equity as one of the major objectives of its latest health reform in 2005 and continues to be a cornerstone indicator of fairness. Previous studies in Chile have demonstrated the relevance of income, level of education and gender on self-reported health outcomes and HRQoL, but none has examined the distribution of HLE by any covariate of socioeconomic status (SES). This scenario motivated the objective of this study, which was to estimate the distribution of health outcomes expressed as LE, QALEs and HALEs by SES, sex and age in the Chilean population to provide a baseline measurement to inform health planning.

## Methods

The present study used four different sources of information. We used data from the National Institute of Statistics (INE, Chile), National Health Survey (ENS 2017), CENSUS (2017) and National Socioeconomic Characterization Survey (CASEN 2017), to calculate the number of populations, number of deaths and weights for HRQoL adjustments according to age, sex and educational level. The data obtained from the INE, and the CENSUS allowed us to construct abbreviated life tables using the Chiang II method. In a second stage, we use the results of the CASEN and ENS 2017 surveys to calculate HALEs and QALEs using the Sullivan´s method. Both the ENS 2017 and CASEN are considered good sources of information to monitor health outcomes and socioeconomic characteristics of the population, because they are nationally representative and contain information about educational level. Finally, we present the distribution of the SMPHs through SES quintiles. Furthermore, we produced estimates of 20:20 absolute and relative gap to show the magnitude of inequalities in a straightforward manner. We argue that these two simple, descriptive inequality results, the graphical description, and the gaps, can be easily explained to policy makers and used for knowledge translation to a lay audience. The 20:20 absolute and relative ratios are often used as a descriptive, crude measure of inequality and it can be found in previous studies [21–23]. Hence, we provide these descriptive findings that are easy to follow and give a general sense of inequality before fully adjusted models are presented and explained.

### Life tables and life expectancy (LE)

Life tables were estimated using the Chiang II´s method [24], using age intervals of 5 years. The method starts estimating the probabilities of dying (*q*_*x*_) for each age interval *x*, as follows:

1 $${q}_{x}=\frac{{n}_{x}{M}_{x}}{1+(1-{a}_{x}){n}_{x}{M}_{x}}$$

Where *n*_*x*_ is the number of years in the age interval *x*; *a*_*x*_ is the proportion of the age interval *x* survived by those dying; and *M*_*x*_ is the mortality rate for the age interval *x*. The *M*_*x*_ parameter was obtained from the INE (INE 2017), and we assumed the usual convention of *M*_*x*_*=1* for the oldest age interval [25]. Then, we simulated a hypothetical cohort of people, who will die over time according to the corresponding probabilities for each age interval. Thus, we estimated the proportion of the cohort alive at the end of the interval (*l*_*x*_), as follows:

2 $${l}_{x}= {l}_{x-1}(1-{q}_{x})$$

In addition, the person years lived in each age interval (*L*_*x*_) was estimated as:

3 $${L}_{x}= {n}_{x}({l}_{x+1}+\left({a}_{x}\left({l}_{x}-{l}_{x+1}\right)\right))$$

which provided the basis to estimate the person years lived in the current and subsequent age intervals (*T*_*x*_), until the oldest (z) interval:

4 $${T}_{x}= \sum _{x=x}^{z}{L}_{x}$$

Finally, the life expectancy (LE), at the beginning of each age interval *x*, was estimated as the quotient between *L*_*x*_ and *T*_*x*_:

5 $${LE}_{x}= \frac{{T}_{x}}{{L}_{x}}$$

### Life Expectancy (LE) by socioeconomic level (SES)

Life tables were built for five quintiles of socioeconomic level (SES), to estimate LE per age interval and SES (*LE*_*x,d*_). Because mortality rates from the INE only provided data about educational level, as the number of years of education, we were only able to implement socioeconomic quintiles for life expectancy using this proxy variable. Thus, we defined five quintiles from the lowest (Q1) to the highest SES (Q5), as follows: Q1 (0–3 years, pre-school), Q2 (3–6 years, early years to year 1); Q3 (6–9 years, primary level); Q4 (9–13 years, secondary level); and Q5 (> 13, technical or university level). Overall, we estimated five life tables, one for each SES quintile, obtaining a distribution of life expectancies across educational level and across age subgroups.

6 $${LE}_{x,d}= \frac{{T}_{x,d}}{{L}_{x,d}}$$

### Quality Adjusted Life Expectancy (QALE)

We applied the Sullivan’s method to produce Quality Adjusted Life Expectancy (QALE). The main source to estimate quality weights was the ENS 2017, which provided answers for the EQ-5D survey in a representative sample of the Chilean population. The survey used the EQ-5D-3L instrument designed by EuroQoL Group, which describes health based on five dimensions of health (mobility, self-care, usual activities, pain or discomfort and anxiety or depression), and a 3 level likert scale to assess the performance of each dimension. Then, using the value sets obtained from the local validation of the instrument EQ-5D-3L in Chile [26], we estimated the specific values, also called utilities, for each age interval *x* and SES subgroup *d*. For consistency, we applied the same definition of quintiles by educational level as mentioned for the LE. In addition, because the ENS 2017 was applied only to adult population (older than 15 years-old) we assumed that population under 15 years-old had the same utility values as the interval 15–20 years-old [25, 27]. Similar to the LE calculation, QALEs at a given age interval is the expected number of quality-adjusted life years (QALY) after that age. Hence, QALEs are calculated as:

7 $${QALE}_{x,d}=\frac{{\varSigma }_{x}^{z}{ L}_{x,d} {u}_{x,d}}{{l}_{x,d}}$$

As shown in Eq. 7, the number of person years lived in the *x* and subsequent z intervals, for one specific SES (*d*), is multiplied by an estimate of the expected utility (*u*_*x,d*_) for the *x* interval and SES. It is worth noting that this utility estimate assumes that HRQoL stays the same over time.

### Health Adjusted Life Expectancy (HALE)

Health-Adjusted Life Expectancy is a SMPH that represents the number of equivalent years with full health one person can expect to live at certain age if he/she was exposed to the prevailing age-specific mortality and morbidity conditions [28, 29]. Its advantage lies on its technical ability to account for the severity of health problems and incorporate information on the specific value of diminished health states compared to perfect health. Alike LE and QALEs, HALE estimated at certain interval *x* is calculated based on survival and the prevalence of discrete health states [30]. Thus, the number of health-adjusted life years in each interval for one specific SES (*Y*_*x,d*_) is obtained multiplying *L*_*x,d*_ by the respective HRQoL weight (*u*_*x,d*_), as follows:

8 $${Y}_{x,d}={L}_{x,d}{\cdot u}_{x,d}$$

And the specific HALE for the *x* age interval and the *d* SES is:

9 $${HALE}_{x,d}=\frac{\sum _{x}^{z}{Y}_{x,d}}{{l}_{x,d}}$$

## Results

The LE of the general population was estimated in 80.4 years, while QALE and HALE at birth were estimated at 69.8 and 62.4, respectively. Women showed LE of 82.9 years, whereas the magnitude for men reached only 77.8 years, which corresponds to a 6.1% lower. Furthermore, QALEs in women and men were estimated in 69.4 and 69.8 respectively, which can be interpreted as to whether there are no a relevant difference. In contrast, we estimated lower magnitudes for HALEs with relevant differences by sex, 60.8 HALEs for women and 64 HALEs for men.

Table 1 shows the national distribution of the three population health summary metrics categorized by the 5 socioeconomic subgroups (defined by years of education) and age intervals. The distribution of LE across the SES presents a consistent gradient in favor of the more educated population regardless the age interval. Although the second (least advantaged) quintile always shows a lower LE than the first education quintile the gradient is very clear from the third quintile onwards.

**Table 1:**
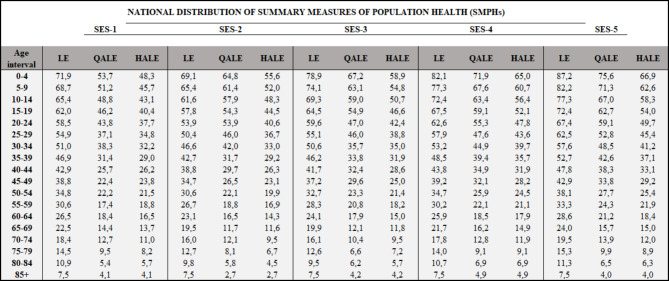
Distribution of summary measures of population health. Life Expectancy (LE), Quality Adjusted Life Expectancy (QALE) and Health Adjusted Life Expectancy (HALE) by socioeconomic status defined by years of education presented in 5-year intervals of age for the Chilean population

**Fig. 1:**
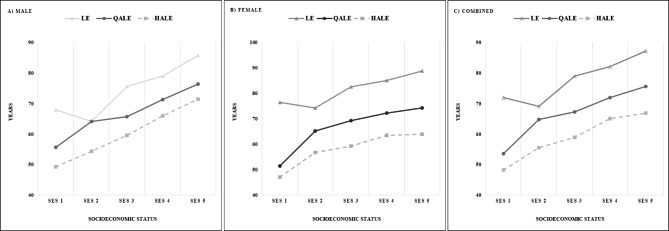
Gradient by socioeconomic status of LE, QALE and HALE at birth for the Chilean population

The quality adjusted metrics also showed a gradient favoring the better educated. Either QALEs or HALEs present a very clear gradient across all quintiles, showing the lowest magnitude in the first quintile and maintained increase up to the most advantaged quintile. This is very consistent in age intervals below 44 years old. The gradients for LE, QALE and HALEs at birth, across quintiles of SES defined by years of education are shown in Fig. 1.

Life expectancy at birth showed an absolute gap of 15.24 years and a relative gap of 1.21, which means one person from the most advantaged education quintile may expect to live 21% longer than another person from the least advantaged quintile. This gap is even larger when we examine HALEs, which reached an absolute gap of 18.57 HALYs and a relative gap of 1.38. These differences are even greater with QALEs, which showed an absolute gap of 21.92 QALYs and a relative gap of 1.41. Therefore, the three metrics show remarkable inequalities in health outcomes in the Chilean population.

**Fig. 2:**
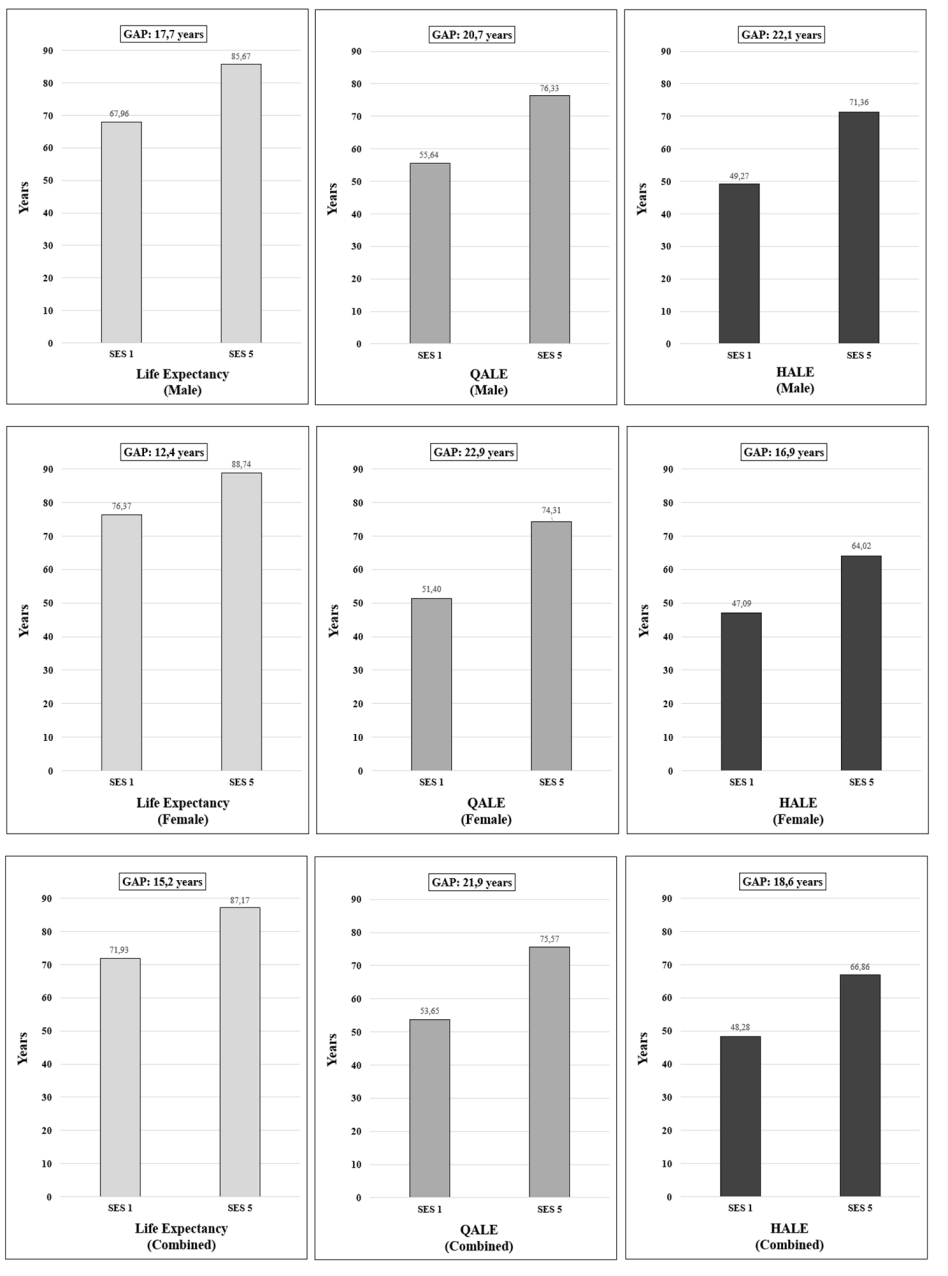
Inequality gaps and predictions of life expectancy (LE), quality-adjusted life expectancy (QALE), and health-adjusted life expectancy (HALE), for population at birth (male, female and combined) in the most and the least deprived socioeconomic quintiles groups

Figure 2 shows the absolute and relative gaps (Q5-Q1) for the general population and categorized by sex. We found a larger absolute health gap in men (17.7 years) compared to women (12.4 years) for LE. Likewise, absolute gaps for HALEs in men were also greater that women (22.1 versus 16.9 HALYs, respectively). QALEs reverts slightly this trend, showing a higher absolute gap in women than men (20.7 versus 22.9 QALYs respectively). Finally, Table 2 shows the absolute and relative gaps for LE, HALE, and QALE for all 5-year intervals of life from birth to 85 years or older.

**Table 2:**
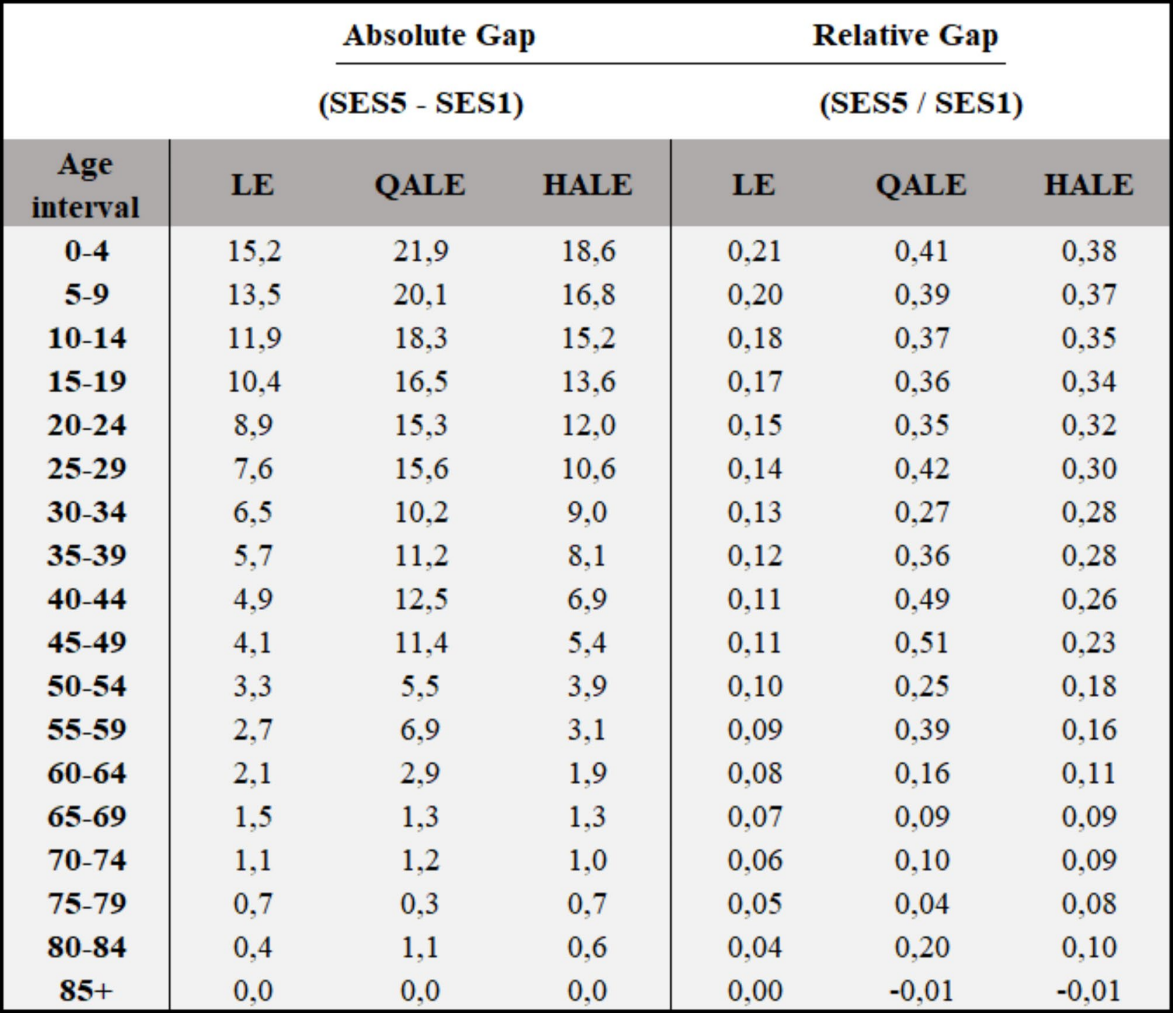
Absolute and Relative inequality gaps (Q5-Q1) of Life Expectancy (LE), Health-Adjusted Life Expectancy (HALE) and Quality-Adjusted Life Expectancy (QALE) and their distribution across 5-years age intervals for the Chilean population

## Discussion

The present study examined the distribution of health outcomes in the general Chilean population through the metrics LE, HALEs and QALEs, across SES, by sex and 5-years age intervals. We found that all three measures showed a clear gradient in favor of the better-off educated population, with gaps up to 21.92 absolute QALY (Q5-Q1) and 1.41 of relative gap (Q5/Q1). In addition, the gaps between the age intervals showed that the greatest inequality is observed at birth, with a decreasing trend over time. The analysis by sex revealed relatively better results for women compared to men in LE, but either QALEs or HALEs showed that men reached slightly better off results. To our knowledge, this is the first study that provides evidence of HLE in Chile and Latin America and, therefore, it represents a baseline for monitoring the overall Chilean population health including HRQoL.

Our study considered educational level as a proxy for evaluating SES, finding an important social gradient between SES categories. That is, the most educated households consistently presented higher levels of LE, QALE and HALE, with some variations among them. The main reason for using years of education as a variable to explore equity issues is its availability from death registries and from the ENS 2017 to estimate HRQoL weights. We chose educational level as the main indicator of SES in this analysis as this variable was consistently available across datasets. For example, household income, another variable frequently used to examine equity concerns, was not available in all datasets for adequate analysis. Additionally, it is well-discussed that household income may not always inform adequately the distributional analysis of SES over the life course. For example, a person may have a low income when he/she dies but has been relatively wealthy for most of his/her life. For this reason (the instability of earnings as a lifetime measure), we decided to conduct our analysis using years of education that are much more stable over time and has significant evidence of correlation with SES [31–33].

One important limitation of the years of education variable is that was trunked in 13 years of education. This means that the highest category may include people with incomplete technical or university education (13 years of education), complete undergraduate studies (14–16 years of education) and people with different types of postgraduate studies (usually greater than 16 years of education). In other words, it is highly likely that the top quintile − 13 or more years of education- contains major socioeconomic differences that we are not able to characterize. On the other hand, it might be criticized that the first two quintiles may be similar in terms of socioeconomic status in the Chilean population. However, we claim that the first quintile is more deprived of basic skills for their social development compared to the second quintile, which is consistent with the gradient observed in our HLE metrics where the first quintile reached systematically lower health outcomes than the second quintile and the following ones.

This study has a number of strengths: (i) it is based on the most recent representative data of the population in Chile, (ii) it uses advanced quantitative methods that allow us to estimate three relevant summary measures of population health that have been recently developed, (iii) it includes a highly accepted and widely used measure of SES (educational level), and (iv) it provides novel reference data on LE, QALE and HALE for South America, in which Chile becomes the case study. However, we also acknowledge some relevant limitations of the study: (i) the cross-sectional nature of the data and the analysis restricts conclusions relating social, political, or economic factors or policies to population health over time; (ii) the limited availability of national registries on mortality only by age, sex and educational level limits our analysis to those indicators only; and (iii) the use of educational level as a single measure of the complex concept of SES.

In terms of educational level, it is widely considered a valid individual indicator of SES, along with income and occupation, and is frequently used as a generic indicator of SES in epidemiological studies. It is thought to capture an individual’s knowledge-related assets being a determinant of future employment and income of individuals. The main advantages of education are that it is relatively easy to measure on self-administered questionnaires, and response rates to educational questions tend to be high. In addition, it can be obtained from anyone regardless of their age or employment circumstances [34, 35]. Currently, in post-industrial societies, education is becoming increasingly important as a “social stratifier”. When the economy becomes dominated by the service sector, as is the case in most European and Western countries, educational credentials partly replace social origins as the “entry ticket” to well-paying jobs and many other social benefits [36, 37]. Educational level is also one of the most stable measure of SES, because it is usually completed early in adulthood, which avoids most reverse causality problems [38, 39]. Educational level has been used in Latin America and the Caribbean [40–42], as well as in other regions [43], proving its usefulness to measure SES and its strong correlation with multiple health outcomes.

On the other hand, it is relevant to point out that the distribution of SES is not only relevant as an outcome but also to implement equity-based economic analysis. More recently, population health distribution has been used to explore the opportunity cost of alternative allocations of health resources in the context of distributional cost-effectiveness analysis [25]. Hence, the information produced in this research does not only support population health monitoring, but also equity informed decisions for coverage of new interventions in healthcare [44, 45]. Given the lack of information of this kind in high-low (like Chile), middle- and low-income countries, we argue that this research may also be relevant in other jurisdictions where this exercise has not been performed yet.

In terms of the reliability of our results, it is important to contrast them with other estimates reported elsewhere. The Global Burden of Disease study (GBD) informs that the LE for Chile were 82.1 and 77.2 years for women and men in 2017, respectively. Those estimates are entirely consistent with our LE estimates (82.7 and 76.4 years for women and men, respectively), which provides evidence of methodological reliability. On the other hand, the same study reported estimations for HALEs in 2017, which were 70.2 and 67.1 years for women and men, respectively. These numbers contrast significantly with our estimates (60.8 and 64 HALYs in women and men, respectively). However, there are important methodological differences between studies. First, the GBD made estimations including quality adjustments associated to each health problem modelled in the global burden of disease exercise. In contrast, our study included quality adjustments by sex and age obtained directly from the health state reports collected in the ENS 2017 through EQ-5D-3L. Second, the GBD used disability weights, which contrasts with the local validated utility weights used in our study. We argue that our approach is better for a local characterization of HALE, whereas the GDB approach can be considered more appropriate for cross country comparisons. Though, it is reasonable to argue that even for international comparisons it should be desirable to produce QALEs and HALEs with local utility weights, to have a better representation of the local social preferences of health. However, this discussion relates with the historical controversy regarding which is a better metric, DALY or QALY [46], which is out of the scope of this research.

We found relevant differences in our main outcomes based on SES. There are several studies exploring the distribution of population health by SES published in the literature [27, 47, 48]. Our study contributes to the consolidation of previous findings that demonstrate a clear gradient of inequalities in favor of better-off educated population [49–51]. However, it also reveals that the magnitude of inequalities in Chile is greater than other high-income countries [27, 52], which may be explained by the more pronounced inequalities in access to the healthcare system but also by more structural social inequalities in Chile [18, 53]. Additionally, the international experience is varied and particularly disparate between low-income and high-income countries, where most reports show large increases in life expectancy in recent decades but without a positive correlation in healthy life expectancy or quality-adjusted life expectancy [54–57]. This challenge presents us with a scenario in which the population is living longer, but disability and poor health occupy an increasing proportion of older people´s lives.

We also found relevant disparities in our main outcomes based on sex. These differences between sex groups in LE and HLE could suggest that women experience significantly higher morbidity than men, impacting on their HRQoL. It could also suggest differentiated responses in self-perceived quality of life between men and women based on gendered expressions of health and disease. For example, there is evidence demonstrating that women in some societies may be more open to express and demand care related to their physical pain and illnesses compared to men [58]. Women may also value quality of life in a different way to men, including informal work, caring for others in their family, and social relations [59, 60]. All of these may be reflected in distinctive gendered patterns of quality of life between men and women. Also, there is a need to expand gender categories in these type of analyses beyond men and women, hence providing a better understanding of gendered patterns among those who are identified in gender and sexual diversity categories. We acknowledge that health inequalities by SES is one of the most important concerns for health policies, though not the only one. Other concerns include gender, geographic location, and ethnicity or belonging to an indigenous population. While we were able to include gender in our analysis, we were not able to examine other concerns because they were not available in the national data sources.

Overall, distribution of health outcomes in Chile shows a clear gradient favoring better-off educated population. In addition, the differences in LE favoring women contrast with estimates of QALEs and HALEs which favor men instead, suggesting that policies to address the morbidity burden should include a gender-informed consideration. Further, population health inequalities across SES are larger in men than women, which is clearer in LE and HALEs, and they decrease over the course of people´s lives. Finally, the magnitude of inequalities in health outcomes found in Chile is larger than what observed in other high-income countries, which can be explained by more structural social inequalities as well as inequalities in access in the healthcare system.

## Data Availability

All data generated or analyzed during this study are included in this published article.

## List of abbreviations

CASEN: Encuesta de caracterización socioeconómica (National Socioeconomic Characterization Survey)
DALY: Disability-adjusted life years
DFLE: Disability-free life expectancy
ENS: Encuesta Nacional de Salud (National Health Survey)
EQ-5D: Euro-Quality of Life – Five Dimensions
HALE: Health- adjusted life expectancy
HALY: Health- adjusted life years
HLE: Healthy life expectancy
HRQoL: Health-Related Quality of Life
INE: Instituto Nacional de Estadisticas (National Institute of Statistics)
LE: Life Expectancy
QALE: Quality-adjusted life expectancy
QALY: Quality-adjusted life years
SES: Socioeconomic Status
SMPH: Summary measure of population health
